# Study on the drug resistance and pathogenicity of *Escherichia coli* isolated from calf diarrhea and the distribution of virulence genes and antimicrobial resistance genes

**DOI:** 10.3389/fmicb.2022.992111

**Published:** 2022-12-22

**Authors:** Yan Jia, Wei Mao, Bo Liu, Shuangyi Zhang, Jinshan Cao, Xiaojing Xu

**Affiliations:** ^1^College of Veterinary Medicine, Inner Mongolia Agricultural University, Hohhot, China; ^2^Key Laboratory of Animal Clinical Treatment Technology, Ministry of Agriculture, Hohhot, China; ^3^Xuzhou Vocational College of Bioengineering, Xuzhou, Jiangsu, China

**Keywords:** *Escherichia coli*, calf diarrhea, drug resistance genes, virulence genes, antimicrobial resistance genes

## Abstract

**Introduction:**

The unscientific and irrational use of antimicrobial drugs in dairy farms has led to the emergence of more serious drug resistance in *Escherichia coli*.

**Methods:**

In this study, cases of calf diarrhea in cattle farms around the Hohhot area were studied, and Escherichia coli were identified by PCR and biochemical methods, while the distribution of virulence and drug resistance genes of the isolates was analyzed.

**Results:**

The results showed that 21 strains of *Escherichia coli* were isolated from the diseased materials, and the isolation rate was 60%. The isolated strains belong to 15 ST types. The drug resistance levels of the isolated strains to 20 kinds of antimicrobial agent viz., penicillin, ampicillin, cefotaxime, cefepime, cefoxitin, and ceftriaxone were more than 50%. The resistance rate to meropenem was 10%. The resistance rates to tetracycline and doxycycline were 33% and 29%, to ciprofloxacin, levofloxacin and enrofloxacin were 48%, 33%, and 33%, to amikacin, kanamycin and gentamicin were 19%, 24% and 38%, to cotrimoxazole and erythromycin were 48% and 15%, to florfenicol, chloramphenicol and polymyxin B were 29%, 33%, and 5%. Nine strains of pathogenic calf diarrhea *Escherichia coli* were isolated by mouse pathogenicity test. The detection rates of virulence genes for the adhesion class were *fimC* (95%), *IuxS* (95%), *eaeA* (76%), *fimA* (62%), *ompA* (52%), and *flu* (24%). The detection rates for iron transporter protein like virulence genes were *iroN* (33%), *iutA* (19%), *fyuA* (14%), *irp5* (9.5%), *Iss* (9.5%), and *iucD* (9.5%). The detection rates for toxin-like virulence genes were *phoA* (90%), *Ecs3703* (57%), *ropS* (33%), *hlyF* (14%), and *F17* (9.5%). The detection rates of tetracycline resistance genes in isolated strains were *tetB* (29%), *tetA* (19%) and *tetD* (14%). The detection rates for fluoroquinolone resistance genes were *parC* (Y305H, P333S, R355G) (9.5%), *gyrA* (S83L, D87N) (28%), *qnrD* (43%), and *qnrS* (9.5%). The detection rates for β-lactam resistance genes were *bla*_*CTX–M*_ (29%), *bla*_*TEM*_ (29%), and bla_*SHV*_ (9.5%). The detection rates for aminoglycoside resistance genes were *strA-B* (57%), *aacC* (33%), *aac(3′)-IIa* (29%), and *aadAI* (24%). The detection rates of chloramphenicol resistance genes *floR* and sulfa resistance genes *sul2* were 24 and 33%.

**Conclusion:**

Pathogenic *Escherichia coli* causing diarrhea in calves contain abundant virulence genes and antibiotic resistance genes.

## Introduction

Calf diarrhea can cause diarrhea in newborn calves and affect normal calf development. This disease is seasonal, with high morbidity and mortality (Hur et al., 2013). A variety of bacteria can cause diarrhea in calves, among which *Escherichia coli* (*E. coli*) is more common. The clinical manifestations are often light brown foul-smelling stools, and later gray watery stools containing bubbles and blood, accompanied by abdominal pain, and occasionally acute toxic cases, which can lead to death or growth retardation (Foster and Smith, 2009).

It is now customary to classify pathogenic *E. coli* as Enterohemorrhagic *E. coli* (EHEC)/Shiga-like toxigenic *E. coli* (STEC), which can secrete characteristic genes such as Shiga toxin (*stx*) and Hemolysin (Melton-Celsa et al., 2012). As well as Enteropathogenic *E. coli* (EPEC) causes adhesion and effacing (A/E) lesions in pathological small intestinal tissues *via* the outer membrane protein adhesion and effacing factor (*eaeA*), which is responsible for encoding a protein located in the enterocyte effacing locus. The EPEC attaching factor (EAF) plasmid encodes the type IV bundle forming pili (*BFP*) gene, which causes pathogenesis in the organism. The atypical EPEC (aEPEC) strain contains only *eaeA* genes. Also, the characteristic genes eaeA and bfp can be identified to determine whether *E. coli* belongs to EPEC (Katsowich et al., 2017). Enterotoxigenic *E. coli* (ETEC) producing enterotoxin can cause disease by producing adhesinogenic hairs *K88*, *K99*, *987P*, *F17*, *F18*, *F41*, etc., and heat-stable enterotoxin (*ST*) and heat-unstable enterotoxin (*LT*) genes acting on intestinal epithelial cells. Whether *E. coli* belongs to ETEC was determined by identifying *STa*, *STb*, *LTI*, and *LTII* subtype genes among *ST* and *LT* signature genes, respectively. Enteric Aggregate *E. coli* (EAEC), Enteroinvasive *E. coli* (EIEC), and Diffusely adherent *E. coli* (DAEC) were identified by aggregative adhesion genes (*aggR*), invasive plasmid antigen H gene (*ipaH*), and Afa/Dr adhesin gene (Robins et al., 2016).

In veterinary practice, antibiotics are often used to treat bacterial calf diarrhea, however, with the continuous and irrational use of antibiotics, the spectrum of *E. coli* resistance is gradually expanding and resistance is becoming increasingly serious. The main mechanisms of drug resistance in *E. coli* include: inactivating or passivating enzymes produced by bacteria that can disrupt the structure of a drug or inhibit its antimicrobial activity (Deckert et al., 2010; Ghotaslou et al., 2014). The mode of bacterial drug resistance gene transmission is mainly plasmid-mediated (Paterson and Bonomo, 2005). The structure of the target of antimicrobial drug action is altered, resulting in the inability of the antimicrobial drug to bind to it (Andrade et al., 2014; Misawa et al., 2018). Bacteria pump antibacterial drugs out of the body by efflux pump (EP) (Fernandez and Hancock, 2013). *E. coli* reduces the accumulation of drugs in cells by altering the permeability of the extracellular membrane (Davies and Davies, 2010). Bacterial biofilm (BF) is regulated by population-sensing (QS)-related genes *luxS*, *mqsR*, regulation of type I hairs (*fimB*), stress regulators (*rpoS*), etc., to make bacteria resistant to drugs (Sharma et al., 2016).

In this study, we analyzed the pathogenicity, drug resistance, strain grouping, and strain sequence type of 21 *E. coli* strains from Hohhot, Inner Mongolia, to provide a test basis for the control of calf diarrhea caused by *E. coli* in the region. The virulence genes and drug resistance genes of the isolated strains were tested to facilitate the understanding of the distribution and prevalence of virulence genes in the sampled herds and the mechanisms of drug resistance of the bacteria in different cattle farms.

## Materials and methods

### Source of strains

Laboratory personnel used disposable samplers to collect anal swabs 35 calves comprising of 26 female and 9 male Holstein calves within 1 month of age with diarrhea symptoms from cattle farms of different sizes around Hohhot, and none of the sampled calves had been given antibiotic treatment. A total of 21 strains of *E. coli*, numbered E1–E21, were isolated from the 35 samples, and *E. coli* ATCC 25922 was kept in our laboratory as a reference strain for subsequent experiments.

### Isolation culture

The 35 samples were first inoculated on normal medium to complete proliferation. Then they were purified and identified by *E. coli* selective medium Eosin-methylene blue medium (EMB) plates and MacConkey Agar (MAC) plates (All the above media were purchased from Qingdao Hi-tech Industrial Park Haibo Biotechnology Co., Qingdao City, China) (Kim et al., 2010).

### Identification

The biochemical reactions were performed on the bacterial solution separately according to the biochemical identification tube instructions. The biochemical characteristics of *E. coli* were positive for maltose, glucose (GLU), mannitol and lactose (MAL), indole, and methyl red (M-R), and negative for acetyl methyl methanol (V-P), Citrate, urea, and hydrogen sulfide (H_2_S) were all negative. The *E. coli*-specific gene 16SrRNA was used as a primer to amplify the isolates. *E. coli* ATCC 25922 was used as the standard strain for all tests in this paper (Kim et al., 2010).

### Multilocus sequence typing of *E. coli*

The housekeeping genes *adk*, *fumC*, *gyrB*, *icd*, *mdh*, *purA*, and *recA* of isolates strains were amplified according to the *E. coli* multilocus sequence typing (MLST) typing scheme provided by the *E. coli* MLST database^1^ (Wirth et al., 2006).

### Drug sensitivity testing

The drug susceptibility testing of isolates E1–E21 was performed by referring to the Kirby-Bauer disk agar diffusion method (K-B) paper diffusion method recommended by the Clinical and Laboratory Standards Institute [CLSI; Weinstein and Lewis (2020)]. The concentration of *E. coli* solution to be tested was adjusted to 1.5 × 10^8^ CFU/ml by turbidimetry, and three replicate groups were made for each strain. The resistance pattern of ATCC 25922 to antibiotics is resistant to ampicillin. ATCC 25922 was used as the control group for the *E. coli* drug susceptibility test, and the criteria for the drug susceptibility test are shown in Supplementary Table 1; Kong et al., 2016.

### Pathogenicity test in mice

The clean-grade mice were randomly divided into 21 test groups and 1 control group, with 4 mice in each group. The 21 test groups of mice were injected intraperitoneally with 0.4 ml of E1–E21 at a concentration of 1.5 × 10^8^ CFU/ml, and the control mice were injected intraperitoneally with an equal amount of saline. The mice were observed continuously for 3 days. The time of onset and death was recorded, and the dead mice were dissected for clinical observation and microscopic examination. The experiments were performed using clean-grade Kunming mice, male and female, weighing about 20–22 g. The mice were purchased from the Animal Experiment Center of Inner Mongolia Medical University, Animal Certificate of Conformity No.: SCXK (Meng) 2020-0001.

### Detection of virulence and resistance genes

Polymerase chain reaction was used to detect the virulence and resistance gene carriage of the isolates, and specific primer sequences were designed for the virulence and resistance gene-specific conserved regions of *E. coli* with reference to the literature. The primer sequences and amplification conditions of *E. coli* virulence and antibiotic resistance genes are shown in Supplementary Tables 2, 3; Paton and Paton, 1998; Kong et al., 1999; Park et al., 2006; Chase et al., 2008; Dallenne et al., 2010; Gündoğdu et al., 2011; Chen et al., 2012; Liu et al., 2016; Belaynehe et al., 2018; Gundran et al., 2019.

## Results

### Results of the identification of *E. coli*

E1–E21 were all consistent with the biochemical characteristics of *E. coli*. The positive control ATCC 25922 and the test samples E1–E21 both amplified specific bands close to the size of the target fragment 16SrRNA (1438 bp). the sequencing results of E1–E21 were compared by BLAST in NCBI and showed more than 99% similarity with the existing sequences in GenBank.

### Results of drug sensitivity tests

**FIGURE 1:**
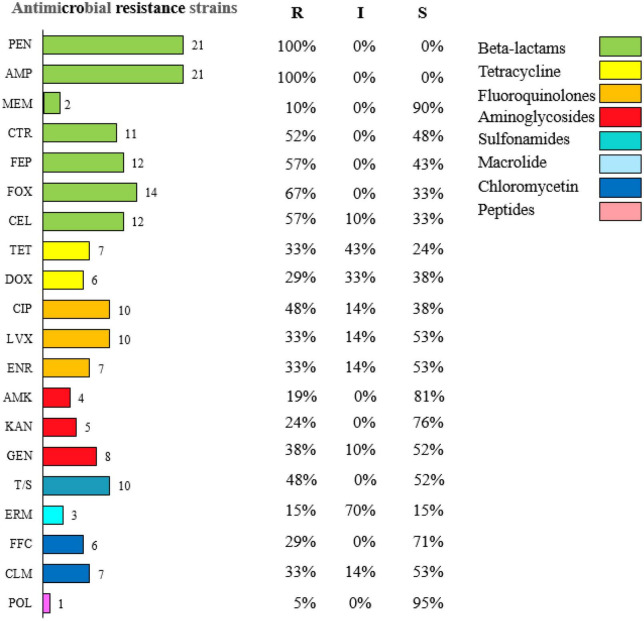
Results of drug sensitivity tests for diarrheagenic calves source *Escherichia coli*.

The resistance test results of E1–E21 could be seen in Figure 1, the highest resistance rate to penicillin and ampicillin was 100%, and the resistance rate to cephalosporins was higher than 50%, but the resistance rate of E1–E21 to meropenem was 10%; The resistance rates to tetracycline and doxycycline were 33 and 29%, to ciprofloxacin, levofloxacin, and enrofloxacin were 48, 33, and 33%, to amikacin, kanamycin, and gentamicin were 19, 24, and 38%, to cotrimoxazole and erythromycin were 48 and 15%, to florfenicol, chloramphenicol, and polymyxin B were 29, 33, and 5%.

### Results of multilocus sequence typing

**FIGURE 2:**
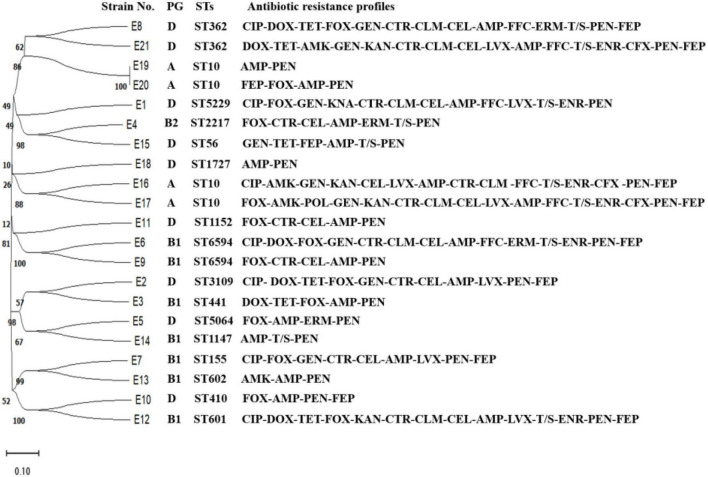
Evolutionary tree of ST types and drug resistance profile in diarrheagenic calves source *Escherichia coli*.

As shown in Figure 2, E1 to E21 were classified into 15 ST types. E4, E16, E17, and E19 were ST10 types, which were the dominant types in this study. E9 and E10 were ST6594 types, E6 and E8 were ST362 types, and the remaining 13 strains of *E. coli* belonged to 13 sequence types, respectively. No new alleles or new sequence types were found.

### Results of pathogenicity tests in mice

The pathogenicity of E1–E21 was identified by a mouse pathogenicity test, and the results showed that E12, E20, E1, E2, E3, E6, E7, E8, and E16 isolates were 100, 100, 50, 75, 25, 25, 25, 75, and 25% lethal to mice, respectively.

### The detection rate of virulence genes of *E. coli* in calves with diarrhea

**FIGURE 3:**
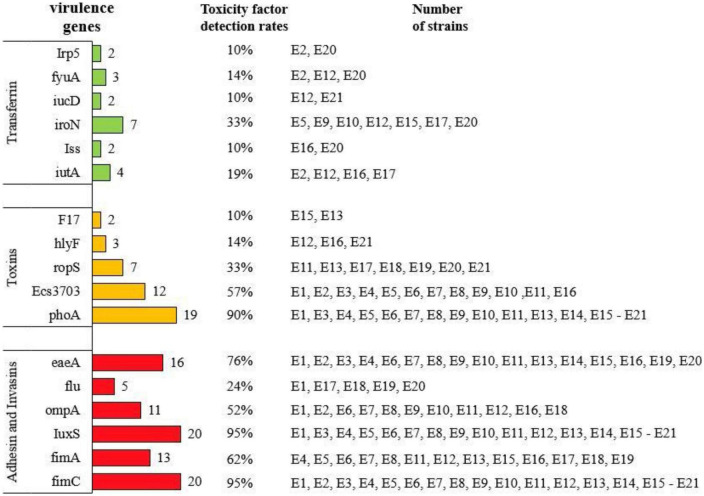
Detection rate of diarrheagenic calves source *Escherichia coli* virulence genes.

As shown in Figure 3, the detection rates of *eaeA*, *fimC*, *fimA*, *IuxS*, *ompA*, and *flu* in the adhesion class of virulence factors from E1 to E21 were 76 (16/21), 95 (20/21), 62 (13/21), 95 (20/21), 52 (11/21), and 24% (5/21), respectively. The detection rates of *fyuA*, *iroN*, *iutA*, *irp5*, *Iss*, and *iucD* among iron transport protein-like virulence factors were 14 (3/21), 33 (7/21), 19 (4/21), 9.5 (2/21), 9.5 (2/21), and 9.5% (2/21), respectively. The detection rates of toxin-like virulence factors *F17*, *hlyF*, *ropS*, *phoA*, and *Ecs3703* were 9.5 (2/21), 14 (3/21), 33 (7/21), 90 (19/21), and 57% (12/21), respectively.

### Association between drug-resistant strains with mutations in the gryA and parC genes and drug resistance

E1, E2, E6, E12, and E21 were all highly resistant to enrofloxacin, levofloxacin, and ciprofloxacin, and only E8 was sensitive to enrofloxacin. All of the above six strains had S83L and D87N double-site mutations in *gyrA*, with E1 and E12 having three point mutations in *parC*, R355G, P333S, and Y305H.

### The detection rate of resistance genes of *E. coli* in calves with diarrhea

**FIGURE 4:**
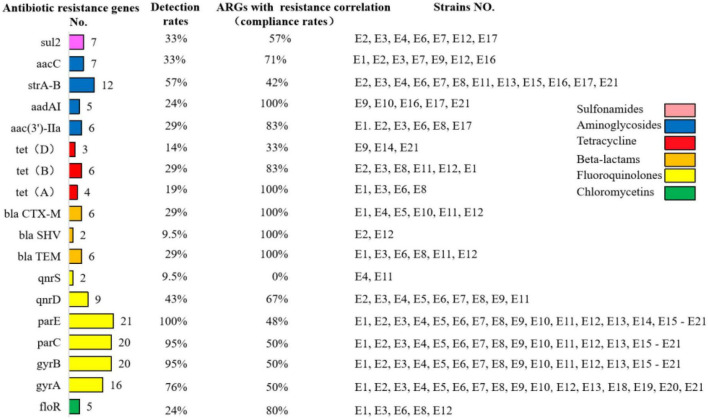
Detection rates of antibiotic resistance genes in diarrheagenic calves source *Escherichia coli* and compliance with correlation with antibiotic resistance correlation.

As shown in Figure 4, the detection rates of tetracycline resistance genes *tetA*, *tetB*, and *tetD* in E1–E21 were 19% (4/21), 29% (6/21), and 14% (3/21), respectively. The detection rates of quinolone resistance genes *gyrA*, *gyrB*, *parC*, *parE*, *qnrD*, and *qnrS* were 76% (16/21), 95% (20/21), 95% (20/21), 100% (21/21), 43% (9/21), and 9.5% (2/21), respectively. The detection rate of mutants of *gyrA* (S83L and D87N) was 28%, and the detection rate of mutants of *parC* (Y305H, P333S, and R355G) was 9.5%. β-lactam resistance genes *bla*_*CTX–M*_, *bla*_*TEM*_, and *bla*_*SHV*_ were detected at 29% (6/21), 29% (6/21), and 9.5% (2/21), respectively. The detection rate of chloramphenicol resistance gene *floR* was 24% (5/21). The detection rate of sul2, a sulfonamide resistance gene, was 33% (7/21). The aminoglycoside resistance genes *aac(3′)-IIa*, *aacC*, *aadAI*, and *strA-B* were detected at 29% (6/21), 33% (7/21), 24% (5/21), and 57% (12/21), respectively.

### Distribution of virulence and drug resistance genes in *E. coli* with calf diarrhea

**FIGURE 5:**
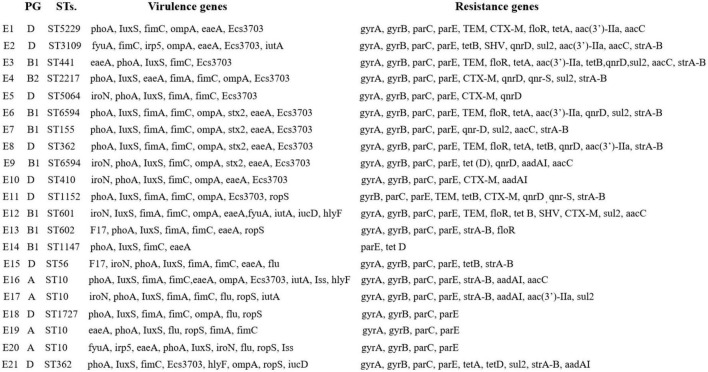
Comparison of virulence and resistance genes in diarrheagenic calves source *Escherichia coli*.

From Figure 5, *eaeA*, *ompA*, *phoA*, *iutA*, *fimA/C*, and *IuxS* are present in groups A, B1, B2, and D. *Ecs3703* and *iroN* are distributed in groups B1, B2, and D in that order, and relatively less in group A. Iss is distributed in group A. hlyF is distributed in groups A and B1. *aadAI*, *aac(3′)-IIa*, *strA-B*, *sul2*, *gyrA*, *gyrB*, *parC*, and *parE* were found in all four groups. *floR* and *qnrD* were distributed in groups B1 and D. The distribution trends of virulence and resistance genes were generally consistent between E1–E21.

### Correlation of antibiotic resistance profiles and resistance genes in calves diarrhea *E. coli*

The compliance rate of resistance among genotype-positive strains in E1–E21 was also positive. The phenotypically resistant antibiotics in the β-lactam class were penicillin, ampicillin, ceftriaxone, cefepime, cefoxitin, and ceftiofur, while their corresponding resistance genes *bla*_*CTX–M*_, *bla*_*TEM*_, and *bla*_*SHV*_ were all 100% consistent with the resistance correlation. The phenotypically resistant antibiotics in the tetracycline class were tetracycline and doxycycline, and the corresponding prevalent resistance genes were *tetA*, *tetB*, and *tetD* with resistance correlations of 100, 83, and 33%, respectively. The phenotypically resistant antibiotics in the chloramphenicol class were florfenicol and chloramphenicol, and the corresponding prevalent resistance genes were *floR* with a resistance correlation rate of 80%. The phenotypically resistant antibiotics in the sulfonamides class were cotrimoxazole, corresponding to the prevalent resistance gene of sul2 and the correlation rate of resistance was 57%. The phenotypically resistant antibiotics among the aminoglycosides were streptomycin, kanamycin, and gentamicin, corresponding to the popular resistance genes *strA-B*, *aac(3′)-IIa*, *aacC*, and *aadAI* with resistance correlation rates of 42, 83, 71, and 100%, respectively. The phenotypically resistant antibiotics in the fluoroquinolones were ciprofloxacin, enrofloxacin, and levofloxacin, corresponding to the prevalent resistance genes *gyrA/B*, *parC/E*, and *qnrD/S* with resistance correlations of 50, 50, 50, 48, 67, and 0%, respectively. The prevalence of resistance and resistance genotypes in the tested strains corresponded to each other; however, due to the limited number of antimicrobials and resistance genes selected in this study, although some resistance genes and resistance phenotypes were more prevalent, it was not possible to determine whether they corresponded to their corresponding resistance phenotypes and resistance genotypes, respectively.

## Discussion

In this study, the results of *in vitro* drug susceptibility tests of 20 antimicrobial drugs from E1–E21 were analyzed for resistance profiles, and it was found that the resistance profiles of the strains varied greatly, with strains that were more than 10 heavy resistance accounted for 38% of the total isolated strains. It can be seen that the current *E. coli* in Hohhot Holstein cattle farm is highly resistant to drugs. The resistance rates of E1–E21 to penicillin and ampicillin were 100%, and the resistance rates to cephalosporins were higher than 50%, however, the resistance rate of E1–E21 to meropenem was 10% similar to the results of domestic and international studies on calf-derived *E. coli* resistance to β-lactam antibiotics (Fazel et al., 2019). E1–E21 were 19, 24, and 38% resistant to amikacin, kanamycin, and gentamicin, respectively, while Algammal found 93.7% resistant to amikacin in calves with *E. coli* in Egypt (Algammal et al., 2020). The resistance rates of E1–E21 to polymyxin B and cotrimoxazole were 5 and 48%, respectively. This finding is consistent with the results of resistance studies on *E. coli* in calves with diarrhea in other countries (Khachatryan et al., 2004). This may be due to the fact that *E. coli* is more susceptible to resistance to sulfonamides. Although multidrug-resistant *E. coli* was shown to be sensitive to polymyxin by Poirel et al. (2017). The resistance rate of E1–E21 to tetracycline was 33%, while Carballo found 66.7% resistance to tetracycline in beef cattle *E. coli* (Carballo et al., 2013). It indicates that the distribution of resistance of various types of antimicrobial drugs to *E. coli* in calf diarrhea at home and abroad showed differences. The resistance to antimicrobial drugs varies among isolates. The epidemiology of drug-resistant *E. coli* in calves may be multifactorial, so veterinarians should use antimicrobial drugs appropriately according to the level of resistance of *E. coli* in different pastures.

In this study, the 21 *E. coli* strains were classified into 15 ST types, indicating that *E. coli* of Hohhot calf origin are characterized by genetic diversity and distant affinity among strains. Because of the limited number of tested strains in this study, it was not possible to accurately describe the association between each ST type category and the germline taxa. Genetic target genes of the corresponding pathogenicity species of IPEC were identified for E1–E21, *eaeA*, and *bfp* genes for EPEC; *STa*, *STb*, and *LTI* and *LTII* genes for ETEC; *aggR* gene for EAEC; *ipaH* gene for EIEC; *Afa/Dr* gene for DAEC; and *stx1* and *stx2* genes for EHEC/STEC. It was found that *eaeA* genes could be detected in 16 strains from E1 to E21, while the representative genes of other pathogenic species were not detected. This indicates that the main prevalent pathogenic type of *E. coli* in calves with diarrhea around the Hohhot area is atypical enteropathogenic *E. coli* (aEPEC).

Only *sul2* (33%) and no *sul1* and *sul3* were detected among the sulfonamide resistance genes in E1–E21, indicating that the *sul2* gene was predominantly prevalent in *E. coli* of Hohhot calf origin, a result consistent with the results of Blahna et al. (2006) who detected *sul2* as the most prevalent gene in *E. coli* of European and Canadian cattle origin. In this study, mobile colistin resistance-1 (*MCR-1*) was not detected which is consistent with the results of Brennan et al. (2016) on the detection rate of *MCR-1* in feces and milk of cattle in France and Germany and Bai et al. (2018) and Zheng et al. (2019) on the carriage rate of *MCR-1* by *E. coli* in dairy farms in several regions of China. The *MCR-1* of dairy-derived *E. coli* showed low levels of prevalence.

In this study, we examined multiple resistance genes contained in ultra broad-spectrum beta-lactamases (ESBLs) and *AmpC* enzymes. *bla*_*CTX–M*_, *bla*_*TEM*_, and *bla*_*SHV*_ were detected at 29, 29, and 9.5%, respectively, and *bla*_*PSE*_, *NDM-1*, *bla*_*OXA*_, and *AmpC* were not detected. It has been shown that ESBLs and *AmpC* enzymes are widely prevalent in *E. coli* of animal origin with the increased frequency of β-lactamases antimicrobial drugs, with *bla*_*CTX–M*_ and *bla*_*TEM*_ types being the more widely distributed genotypes at present (Tangden et al., 2010; Gautam et al., 2019).

E1 to E21 carried *tetA* (19%), *tetB* (29%), and *tetD* (14%), and *tetC* and *tetE* were not detected. *tetA* (35%) and *tetB* (63%) were found to be the main prevalent resistance genes for tetracycline in different animal organisms of *E. coli* by Shapir et al. (2004). This may be due to the different prevalence of resistance genes in cows versus beef cattle. *gyrA* (76%), *gyrB* (95%), *parC* (95%), *parE* (100%), *qnrD* (43%), and *qnrS* (9.5%) were carried in E1–E21, and *qnrA/B/C*, *oqxA/B*, and *qepA* were not detected. Detection of these genes does not necessarily imply that they will exhibit resistance, as these genes may remain unexpressed in bacteria (Olonitola et al., 2015). No NDM-related genes were detected in ST410 *E. coli* in this study, but it carried drug resistance genes such as *gyrA*, *gyrB*, *parC*, *parE*, *CTX-M*, and *aadAI* (Gamal et al., 2016). In this study, amino acid substitutions in S83L and D87N were found to occur in the Quinolone-Resistant Determining Region (QRDR) region encoded by the *gyrA* gene in six strains, and amino acid substitutions in R355G, P333S, and Y305H were found for the first time in the *parC* subunit. It has been shown earlier that double mutation sites (S83L and D87N, D87G, or D87Y) exist in *gyrA*, while S80I, S80R, or E84K single point mutations exist in *parC* and S459A or N463D single point mutations are found in *parE* (Babus et al., 1998; Piddock, 1999). The amino acid substitution site of *gyrA* found in this study was consistent with related reports from home and abroad, while the type of amino acid substitution on the *parC* subunit was found for the first time.

The resistance and resistance gene carriage of E1–E21 showed roughly positive correlation, such as kanamycin, gentamicin, and amikacin with resistance genotypes *aac(3′)-IIa*, *aacC*, and *aadAI* conformed to 83, 71, and 100%, respectively. Streptomycin had 42% concordance with resistance genotype *strA-B*; tetracycline and doxycycline had 100, 83, and 33% concordance with resistance genotype *tetA/B/D*, respectively; florfenicol and chloramphenicol had 80% concordance with resistance genotype *floR*; cotrimoxazole had 57% concordance with resistance genotype *sul2*; penicillin, ampicillin, and cephalosporins had 100% concordance with resistance *bla*_*CTX–M*_, *bla*_*SHV*_, and *bla*_*TEM*_ were all 100%; ciprofloxacin, levofloxacin, and enrofloxacin were 50, 50, 50, 48, 67, and 0%, respectively with the resistant genotypes *gyrA/B*, *parC/E*, and *qnrD/S*. The strains with amino acid substitutions in the *parC* and *gyrA* subunits in this test showed severe resistance to enrofloxacin, levofloxacin, and ciprofloxacin. The amino acid substitutions on the *parC* and *gyrA* subunits of strains with the same resistance phenotype varied greatly. It has been suggested that carrying the corresponding resistance genes may lead to the development of corresponding drug resistance (Han et al., 2013). The lower detection rate of genes encoding β-lactam resistance than phenotypic resistance in this study may be attributed to the fact that other β-lactamase genes were not studied or other resistance mechanisms were not addressed.

## Data availability statement

The original contributions presented in this study are included in this article/Supplementary material, further inquiries can be directed to the corresponding authors. The data presented in the study are deposited in the GenBank Overview (nih.gov) repository, accession number 2654460.

## Ethics statement

All animal experiments were performed according to regulations of the Administration of Affairs Concerning Experimental Animals in China. The experimental protocol was approved by the Animal Welfare and Research Ethics Committee of the Inner Mongolia Agricultural University (Approval ID: NND202103).

## Author contributions

WM, BL, SZ, XX, and JC: preparation, creation, presentation of the published work, and specifically writing the initial draft (including substantive translation). YJ: methodology, data analysis, and original draft. All authors contributed to the article and approved the submitted version.

## Footnotes

1
http://shigatox.net/ecmlst/cgi-bin/dbquery
