# Supplementary material for: Study on the drug resistance and pathogenicity of Escherichia coli isolated from calf diarrhea and the distribution of virulence genes and antimicrobial resistance genes
Source: Front Microbiol. 2022 Dec 22;13:992111. doi: 10.3389/fmicb.2022.992111 (PMC9815963; doi:10.3389/fmicb.2022.992111)
Supplement: Supplementary file 1 [file Table_1.docx]

Table. 1 Criteria for drug sensitivity test of *E. coli*

| Types of drugs | Antibiotics | Drug contents（μg/ piece） | Sensitivity Decision Folders /（mm） | | | ATCC-25922 |
| --- | --- | --- | --- | --- | --- | --- |
|  |  | Diameter 6mm/ piece | S | I | R |  |
| β-lactams | Meropenem（MEM） | 10μg | ≥23 | 20-22 | ≤19 | 28-35 |
|  | Ampicillin（AMP） | 10μg | ≥17 | 14-16 | ≤13 | 15-22 |
|  | Penicillin（PEN） | 10U | ≥18 | 14-17 | ≤13 | — |
|  | Cephalothin（CEL） | 30μg | — | — | — | 15-21 |
|  | Cefepime（FEP） | 30μg | ≥25 | — | ≤18 | 31-37 |
|  | Cefoxitin（FOX） | 30μg | ≥18 | 15-17 | ≤14 | 23-29 |
|  | Ceftriaxone（CTR） | 30μg | ≥23 | 20-22 | ≤19 | 29-35 |
| Tetracyclines | Doxycycline（DOX） | 30μg | ≥14 | 11-13 | ≤10 | 18-24 |
|  | Tetracycline（TET） | 30μg | ≥15 | 12-14 | ≤11 | 18-25 |
| Fluoroquinolones | Ciprofloxacin（CIP） | 5μg | ≥26 | 22-25 | ≤21 | 29-37 |
|  | Levofloxacin（LVX） | 5μg | ≥21 | 17-20 | ≤16 | 29-37 |
|  | Enrofloxacin（ENR） | 5μg | ≥18 | 15-17 | ≤14 | — |
| Aminoglycosides | Amikacin（AMK） | 30μg | ≥17 | 15-16 | ≤14 | 19-26 |
|  | Kanamycin（ANA） | 30μg | ≥18 | 14-17 | ≤13 | 17-25 |
|  | Gentamicin（GEN） | 10μg | ≥15 | 13-14 | ≤12 | 19-26 |
| Sulfonamides | Cisplatin（T/S） | 23.75μg | ≥16 | 11-15 | ≤10 | — |
| Macrolides | Erythromycin（ERM） | 15μg | — | — | — | — |
| Chloramphenicol | Chloromycin（CLM） | 30μg | ≥18 | 13-17 | ≤12 | 21-27 |
|  | Florfenicol（FFC） | 30μg | ≥18 | 13-17 | ≤12 | — |
| Polypeptides | Polymyxin B（POL） | 300U | ≥12 | 9-11 | ≤8 | 13-19 |

Note: Sensitivity (S); Intermerdiary (I); Resistance (R).

Table. 2 Primer sequences and amplification conditions of *E. coli* virulence genes

| Gene | Primer sequences (5’→3’) | | | Length（bp） | Denaturing | Annealing | Extension |
| --- | --- | --- | --- | --- | --- | --- | --- |
| irp2 | F: AGGATTCGCTGTTACCGGAC  R: TCGTCGGGCGGCGTTTCTTC | | | 286 bp | 94℃for  30sec | 60℃for  25sec | 72℃for  20sec |
| fyuA | F:TGATTAACCCCGCGACGGGAA  F:CGCAGTAGGCACGATGTTGTA | | | 880 bp | 94℃for  30sec | 60℃for  25sec | 72℃for  50sec |
| Iss | F: CCGTTCTGTACGATACTCCG  R: AGATCAATCTGCCCATC | | | 971 bp | 94℃for  30sec | 60℃for  25sec | 72℃for  50sec |
| iucD | F: GCGGAAATAAACTGGGTC  R: GGCATCAGTGAGGGAAGTA | | | 410 bp | 94℃for  30sec | 60℃for  25sec | 72℃for  30sec |
| irp5 | F:CGCGGATCCGGTACCAGGTGACGCATGAT  R: AACTGCAGTCAACCTGTTTCGGGTCGG | | | 1636 bp | 94℃for  30sec | 60℃for  30sec | 72℃for  95sec |
| iroN | F: AATCCGGCAAAGAGACGAACCGCCT  R: GTTCGGGCAACCCCTGCTTTGACTTT | | | 553 bp | 94℃for  30sec | 55℃for  25sec | 72℃for  30sec |
| iutA | F: GGCTGGACATCATGGGAACTGG  R: CGTCGGGAACGGTAGAATCG | | | 300 bp | 94℃for  30sec | 54℃for  25sec | 72℃for  30sec |
| F41 | F:GCATCAGCGGCAGTATCT  R: GTCCCTAGCTCAGTATTATCACCT | | | 380 bp | 94℃for  30sec | 50℃for  45sec | 72℃for  25sec |
| K88 | F: GCTGCATCTGCTGCATCTGGTATG  R: CCACTGAGTGCTGGTAGTTACAGCC | | | 792 bp | 94℃for  30sec | 60℃for  25sec | 72℃for  50sec |
| K99 | F:TATTATCTTAGGTGGTATGG  R: GGTATCCTTTAGCAGCAGTATTTC | | | 314 bp | 94℃for  30sec | 56℃for  25sec | 72℃for  25sec |
| F17 | F: GCAGAAAATTCAATTTATCCTTGG  R: CTGATAAGCGATGGTGTAATTAAC | | | 537 bp | 94℃for  60sec | 57℃for  60sec | 72℃for  35sec |
| F18 | F: GTGAAAAGACTAGTGTTTATTTC  R: CTTGTAAGTAACCGCGTAAGC | | | 510 bp | 94℃for  30sec | 55℃for  25sec | 72℃for  30sec |
| 987P | F: TCTGCTCTTAAAGCTACTGG  R: AACTCCACCGTTTGTATCAG | | | 333 bp | 94℃for  30sec | 56℃for  25sec | 72℃for  30sec |
| espA | F: TATCAGGCACAAAGCGATCTGTC  R: TATCTCCGGTTATTTACCAAGGG | | | 432 bp | 94℃for  30sec | 55℃for  25sec | 72℃for  30sec |
| flu | F: CTGGTATGGAATCACTTACGGG  R: GAGAATGCTCCCAGGCGGTTTAT | | | 965 bp | 94℃for  30sec | 57℃for  30sec | 72℃for  60sec |
| ompA | F: ACGCTGTTTCACGTTGTCA  R: AACCCGTATGTTGGCTTTG | | | 753 bp | 94℃for  30sec | 55℃for  25sec | 72℃for  45sec |
| ompT | F: TCATCCCGGAAGCCTCCCTCACTACTAT  R: TAGCGTTTGCTGCACTGGCTTCTGATAC | | | 496 bp | 94℃for  30sec | 57℃for  35sec | 72℃for  30sec |
| IuxS | F: ATGCCGTTGTTAGATAGC  R: CTAGATGTGCAGTTCCTGC | | | 516 bp | 94℃for  30sec | 55℃for  25sec | 72℃for  30sec |
| fimA | F:GCTCTGGCTGATACTACACC  R: TTATTGATACTGAACCTTGA | | | 495 bp | 94℃for  30sec | 58℃for  25sec | 72℃for  30sec |
| fimC | F: GTTCATGGCAATGGTGGTT  R: AGTTCCGGCATTCAACTCT | | | 514 bp | 94℃for  30sec | 60℃for  25sec | 72℃for  30sec |
| eaeA | F: CTGAACGGCGATTACGCGAA  R: CCAGACGATACGATCCAG | | | 798 bp | 94℃for  30sec | 52℃for  30sec | 72℃for  45sec |
| ler | F: AACAAGCCCATACATTCAGC  R: GCCATCATCAGGCACATTAG | | | 169 bp | 94℃for  30sec | 55℃for  25sec | 72℃for  20sec |
| LT-I | F: GGCGACAGATTATACCGTGC  R: CGGTCTCTATATTCCCTGTT | | | 450 bp | 94℃for  30sec | 54℃for  25sec | 72℃for  30sec |
| LT-II | F: AGATATAATGATGGATATGTATC  R: TAACCCTCGAAATAAATCTC | | | 300 bp | 94℃for  30sec | 53℃for  25sec | 72℃for  25sec |
| STa | F:GCTAATGTTGGCAATTTTTATTTCTGTA  R: AGGATTACAACAAAGTTCACAGCAGTAA | | | 190 bp | 94℃for  30sec | 50℃for  45sec | 72℃for  35sec |
| STb | F:GCAATAAGGTTGAGGTGAT  R: TGTTGTACGAAATCCCCTCTG | | | 368 bp | 94℃for  30sec | 60℃for  25sec | 72℃for  25sec |
| Stx1 | F: TTCGCTCTGCAATAGGTA  R: TTCCCCAGTTCAATGTAAGAT | | | 555 bp | 94℃for  30sec | 54℃for  45sec | 72℃for  35sec |
| Stx2 | F: CCATGACAACGGACAGCAGTT  R: CCTGTCAACTGAGCAGCACTTTG | | | 779 bp | 94℃for  30sec | 55℃for  45sec | 72℃for  45sec |
| hlyE | F: CTCAATCGGCATCCACAT  R: GTTTCCCTTCAACAACCC | | | 456 bp | 94℃for  30sec | 52℃for  35sec | 72℃for  30sec |
| hlyA | F: GCATCATCAAGCGTACGTTCC  R: AATGAGCCAAGCTGGTTAAGCT | | | 534 bp | 94℃for  30sec | 60℃for  45sec | 72℃for  35sec |
| hlyF | F:TGGCCACAGTCGTTTAGGGTGCTTACC  R: GGCGGTTTAGGCATTCCGATACTCAG | | | 450 bp | 94℃for  30sec | 58℃for  25sec | 72℃for  30sec |
| phoA | F: CGATTCTGGAAATGGCAAAAG  R: CGTGATCAGCGGTGACTATGAC | | | 720 bp | 94℃for  30sec | 55℃for  25sec | 72℃for  45sec |
| ipaH | F: GTTCCTTGACCGCCTTTCCGATACCGTC  R: GCCGGTCAGCCACCCTCTGAGAGTAC | | | 619 bp | 94℃for  30sec | 55℃for  25sec | 72℃for  35sec |
| aggR | | F: GTATACACAAAAGAAGAAGGAAGC  R:ACAGAATCGTCAGCATCAGC | | 254 bp | 94℃for  30sec | 55℃for  25sec | 72℃for  20sec |
| pfs | F: ATGAAAATCGGCATCATT  R: TTAGCCATGTGCAAGTTT | | | 699 bp | 94℃for  30sec | 60℃for  25sec | 72℃for  45sec |
| ropS | F: TACCTTGGTGAGATTGGTATTC  R: ATTTCACGACCTACATCTTCCAG | | | 704 bp | 94℃for  30sec | 55℃for  25sec | 72℃for  30sec |
| TraT | F: GGTGTGGTGCGATGAGCACAG  R: CACGGTTCAGCCATCCCTGAG | | | 290 bp | 94℃for  30sec | 55℃for  30sec | 72℃for  20sec |
| Ecs3703 | | | F: CATGCAATAGTTGCTCAATGC  R: CCCATTCTCTTTTCGATTCG | 552 bp | 94℃for  30sec | 55℃for  25sec | 72℃for  30sec |
| aer | F: TACCGGATTGTAATATGCAGACCGT  R: AATATCTTCCTCCAGTCCGGAGAAG | | | 602 bp | 94℃for  30sec | 55℃for  25sec | 72℃for  35sec |

Note: F represents the upstream primer; R represents the downstream primer.

Table. 3 Primer sequences and amplification conditions of *E. coli* Antimicrobial resistance genes

| Gene | Primer sequences (5’→3’) | | | | Length（bp） | Denaturing | annealing | | extension | |
| --- | --- | --- | --- | --- | --- | --- | --- | --- | --- | --- |
| gyrA | F: GGTGACGTAATCGGTAAATA  R: ACCATGGTGCAATGCCACCA | | | | 810 | 94℃for  30sec | 53℃for  45sec | | 72℃for  35sec | |
| gyrB | F: GGACAAAGAAGGCTACAGCA  F: CGTCGCGTTGTACTCAGATA | | | | 879 | 94℃for  30sec | 53℃for  25sec | | 72℃for  50sec | |
| parC | F: CTGGGTAAATACCATCCGCAC  R: CGGTTCATCTTCATTACGAA | | | | 987 | 94℃for  30sec | 55℃for  25sec | | 72℃for  60sec | |
| parE | F: CTGACCGAAAGCTACGTCAACC  R: CGTTCGGCTTGCCTTTCTTG | | | | 892 | 94℃for  30sec | 55℃for  30sec | | 72℃for  50sec | |
| qnrA | F: ATTTCTCACGCCAGGATTTG  R: GATCGGCAAAGGTCAGGTCA | | | | 516 | 94℃for  30sec | 56℃for  25sec | | 72℃for  30sec | |
| qnrB | F: GATCGTGAAAGCCAGAAAGG  R: ACGATGCCTGGTAGTTGTCC | | | | 469 | 94℃for  30sec | 53℃for  25sec | | 72℃for  30sec | |
| qnrC | F: ATTTCTCACAGGCAAACT  R: CTGGAATAACAATCACCC | | | | 660 | 94℃for  30sec | 56℃for  25sec | | 72℃for  40sec | |
| qnrD | F: TTTTCGCTAACTAACTCGC  R: GAAAGGATAAACAGGCAAAT | | | | 984 | 94℃for  30sec | 56℃for  25sec | | 72℃for  60sec | |
| qnrS | F: GCAAGTTCATTGAACAGGGT  R: TCTAAACCGTCGAGTTCGGCG | | | | 428 | 94℃for  30sec | 52℃for  25sec | | 72℃for  30sec | |
| oqxB | F: TTCTCCCCCGGCGGGAAGTAC  R: CTCGGCCATTTTGGCGCGTA | | | | 512 | 94℃for  30sec | 55℃for  25sec | | 72℃for  30sec | |
| _bla_TEM | | F: GTATCCGCTCATGAGACAATA  R: AGAAGTGGTCCTGCAACTTT | | | 717 | 94℃for  30sec | | 53℃for  30sec | | 72℃for  45sec |
| _bla_SHV | | F: ATGCGTTATATTCGCCTGTG  R: TTAGCGTTGCCAGTGCTCGA | | | 860 | 94℃for  30sec | | 56℃for  25sec | | 72℃for  50sec |
| _bla_CTX-M | | | F: GGTGTGGTGCGATGAGCACAG  R: CACGGTTCAGCCATCCCTGAG | | 544 | 94℃for  30sec | | 55℃for  25sec | | 72℃for  30sec |
| _bla_PSE | F: CCCTTCGGGTTAACAAGTAC  R: CTGGTTCATTTCAGATAGCG | | | | 419 | 94℃for  30sec | 55℃for  25sec | | 72℃for  30sec | |
| NDM-1 | F: TCGCCCCATATTTTTGCTACAG  R: CGATCCTTCCAACTCGTCGC | | | | 800 | 94℃for  30sec | 55℃for  25sec | | 72℃for  30sec | |
| _bla_OXA | F: TTTTCTGTTGTTTGGGTTTC  R: TTTCTTGGCTTTTATGCTTG | | | | 447 | 94℃for  30sec | 55℃for  25sec | | 72℃for  30sec | |
| ampC | F: CCCCGCTTATAGAGCAACAA  R: TCAATGGTCGACTTCACACC | | | | 634 | 94℃for  60sec | 55℃for  35sec | | 72℃for  35sec | |
| floR | | F: GAACACGACGCCCGCTAT  R: TTCCGCTTGGCCTATGAG | | | 601 | 94℃for  30sec | | 55℃for  25sec | | 72℃for  35sec |
| cat | | F:AGTTGCTCAATGTACCTATAACC  R:TTGTAATTCATTAAGCATTCTGCC | | | 547 | 94℃for  60sec | | 55℃for  30sec | | 72℃for  30sec |
| cmlA | | F: CCGCCACGGTGTTGTTGTTATC  R: CACCTTGCCTGCCCATCATTAG | | | 698 | 94℃for  30sec | | 55℃for  30sec | | 72℃for  40sec |
| tet A | | F: GCTACATCCTGCTTGCCTTC  R: CATAGATCGCCGTGAAGAGG | | | 210 | 94℃for  30sec | | 58℃for  25sec | | 72℃for  20sec |
| tet B | | F: TTGGTTAGGGGCAAGTTTTG  R: GTAATGGGCCAATAACACCG | | | 659 | 94℃for  30sec | | 58℃for  25sec | | 72℃for  40sec |
| tet C | | F:CTTGAGAGCCTTCAACCCAG  R: ATGGTCGTCATCTACCTGCC | | | 418 | 94℃for  30sec | | 58℃for  25sec | | 72℃for  30sec |
| tet E | | F: AAACCACATCCTCCATACGC  R: AAATAGGCCACAACCGTCAG | | | 278 | 94℃for  30sec | | 58℃for  30sec | | 72℃for  25sec |
| tet D | | F: AAACCATTACGGCATTCTGC  R: GACCGGATACACCATCCATC | | | 787 | 94℃for  30sec | | 58℃for  25sec | | 72℃for  45sec |
| sul1 | | F: GTGACGGTGTTCGGCATTCT  R: TCCGAGAAGGTGATTGCGCT | | | 779 | 94℃for  30sec | | 63℃for  30sec | | 72℃for  45sec |
| sul2 | | F: CGGCATCGTCAACATAACCT  R: TGTGCGGATGAAGTCAGCTC | | | 721 | 94℃for  30sec | | 57℃for  25sec | | 72℃for  45sec |
| Sul3 | | F: GAGCAAGATTTTTGGAATCG  R:CATCTGCAGCTAACCTAGGCTTTGGA | | | 880 | 94℃for  30sec | | 55℃for  30sec | | 72℃for  50sec |
| strA-B | F:TATCTGCGATTGGACCCTCTG  R: CATTGCTCATCATTTGATCGGCT | | | | 538 | 94℃for  30sec | | 55℃for  25sec | | 72℃for  30sec |
| aac（3’）-IIa | | | | F: GGCGACTTCACCGTTTCT  R: GGACCGATCACCCTACGAG | 412 | 94℃for  30sec | | 54℃for  25sec | | 72℃for  30sec |
| aac（6’）-Ib | | | | F: TTGCGATGCTCTATGAGTGGCTA  R: CTCGAATGCCTGGCGTGTTT | 482 | 94℃for  30sec | | 55℃for  25sec | | 72℃for  30sec |
| aadB | F:GAGGAGTTGGACTATGGATT  R: CTTCATCGGCATAGTAAAA | | | | 208 | 94℃for  30sec | | 53℃for  30sec | | 72℃for  20sec |
| aadAI | F: GCAGCGCAATGACATTCTTG  R: ATCCTCGGCGCGATTTTG | | | | 282 | 94℃for  30sec | | 53℃for  30sec | | 72℃for  20sec |
| aacC | F: ACCCTACGAGGAGACTCTGAATG  R: CCAAGCATCGGCATCTCATA | | | | 384 | 94℃for  30sec | | 55℃for  25sec | | 72℃for  25sec |
| aphA | F: TGACTGGGCACAACAGACAA  R: CGGCGATACCGTAAAGCAC | | | | 697 | 94℃for  30sec | | 55℃for  25sec | | 72℃for  40sec |
| mcr-I | F: GCTCGGTCAGTCCGTTTGTTC  R:CGGCGTTTAATAGGATCCTTGGTCTCG | | | | 338 | 94℃for  30sec | | 55℃for  25sec | | 72℃for  25sec |

Note: F represents the upstream primer; R represents the downstream primer.
